# Proximal Humerus Internal Locking System (PHILOS) Plating Versus External Fixation Using Kirschner Wires (K-Wires) With Frames for Proximal Humerus Fractures: A Prospective Comparative Study

**DOI:** 10.7759/cureus.112544

**Published:** 2026-07-12

**Authors:** Pavneesh Kumar, Indrajit D Bhoumik, Pearly Swedia, Anil K Joshi, Aditya K Singh

**Affiliations:** 1 Department of Orthopedics, Government Doon Medical College, Dehradun, IND

**Keywords:** external fixation, k-wire fixation, philos plating, proximal humerus fracture, quickdash

## Abstract

Background: Proximal humerus fractures are among the most common fractures involving the shoulder and frequently require surgical intervention when displaced or unstable. Among the available fixation techniques, open reduction and internal fixation using proximal humerus internal locking system (PHILOS) plates and external fixation using Kirschner wires (K-wires) and frames are commonly employed. However, evidence comparing their clinical and functional outcomes remains limited, particularly in the Indian population.

Methods: This prospective comparative observational study was conducted at Government Doon Medical College and associated Doon Hospital, Dehradun, India, over a period of 15 months. Sixty adult patients with proximal humerus fractures were included: 30 treated with PHILOS plating (MEDPLUS, Ahmedabad, India) and 30 with external fixation using K-wires and frames (MEDPLUS, Ahmedabad, India). Baseline demographic, clinical, and fracture characteristics were recorded. Functional outcomes were assessed using the Quick Disabilities of the Arm, Shoulder and Hand (QuickDASH) score at one, three, and six months postoperatively. Secondary outcomes included operative duration, intraoperative blood loss, fluoroscopy exposure, hospital stay, time to radiological union, shoulder range of motion, and complications.

Results: Radiological union rates were comparable between groups. By 16 weeks, union was achieved in 27/30 patients (90.0%) in the PHILOS group and 25/30 patients (83.3%) in the K-wires with frames group; no patient in either group developed non-union. Functional outcomes favored PHILOS plating. QuickDASH scores were significantly lower at three months (31.80 ± 9.88 vs. 37.60 ± 8.11, p = 0.016) and six months (16.89 ± 8.48 vs. 22.59 ± 8.54, p = 0.012). Patients treated with PHILOS plating also demonstrated significantly greater forward flexion (142.60 ± 18.40° vs. 131.76 ± 21.65°, p = 0.041) and abduction (136.87 ± 20.15° vs. 124.70 ± 22.51°, p = 0.031) at six months. Repeated-measures analysis demonstrated significant improvement in QuickDASH scores over time, F(1.565, 90.770) = 1958.187, p <0.001, and significantly lower overall QuickDASH scores in the PHILOS plating group, F(1, 58) = 5.774, p = 0.019; however, the time × treatment group interaction was not significant. Overall complication rates were identical, occurring in 8/30 patients (26.7%) in each group. Fracture complexity, comorbidities, and postoperative complications were significantly associated with poorer functional outcomes.

Conclusions: Both PHILOS plating and external fixation using K-wires with frames achieved satisfactory fracture union and functional recovery. However, patients treated with PHILOS plating demonstrated better functional outcomes, earlier painless mobilization, and improved shoulder range of motion despite longer operative time, greater blood loss, and prolonged hospitalization. PHILOS plating may therefore be the preferred treatment option for appropriately selected proximal humerus fractures.

## Introduction

Proximal humerus fractures account for approximately 4-6% of all fractures and are among the most common injuries involving the shoulder joint [1,2]. These fractures affect the proximal portion of the humerus, including the humeral head, greater tuberosity, lesser tuberosity, and surgical neck. Because these structures contribute to shoulder stability and serve as attachment sites for the rotator cuff muscles, fracture displacement can significantly impair shoulder function, resulting in pain, weakness, restricted range of motion, and reduced quality of life [1,3].

The Neer classification system is widely used to categorize proximal humerus fractures based on the number of displaced segments. Fractures are classified as one-part, two-part, three-part, or four-part injuries, with increasing complexity associated with greater instability, higher complication rates, and poorer functional outcomes. This classification remains an important tool for treatment planning and predicting prognosis [4].

The incidence of proximal humerus fractures has increased worldwide due to ageing populations and the rising prevalence of osteoporosis. These injuries show a bimodal distribution, occurring in elderly individuals after low-energy falls and in younger adults following high-energy trauma such as road traffic accidents or falls from height. In India, trauma-related proximal humerus fractures constitute a substantial proportion of orthopedic admissions and frequently require surgical management [2,5].

Treatment depends on fracture pattern, degree of displacement, bone quality, and patient functional demands. While minimally displaced fractures can often be treated conservatively, displaced and unstable fractures usually require surgical fixation to restore anatomy, achieve fracture union, and allow early mobilization [3,6]. Among the available surgical options, open reduction and internal fixation using the proximal humerus internal locking system (PHILOS) plate has become a widely accepted method. PHILOS plates provide angular-stable fixation and maintain reduction even in osteoporotic bone, facilitating early rehabilitation and functional recovery [7]. However, complications such as screw penetration, varus collapse, hardware-related problems, and avascular necrosis have been reported [8,9].

External fixation using percutaneous Kirschner wires (K-wires) connected to an external frame is a less invasive alternative that aims to preserve soft tissues and fracture biology [10]. By minimizing surgical exposure and periosteal disruption, this technique may reduce blood loss and preserve vascularity of the humeral head. Nevertheless, concerns remain regarding its mechanical stability, particularly in complex fracture patterns [11].

Despite advances in implant design and surgical techniques, the optimal fixation method for displaced proximal humerus fractures remains controversial [12,13]. Locking plate fixation offers superior mechanical stability, whereas external fixation emphasizes biological preservation and minimal soft tissue damage. Current evidence comparing these techniques remains limited, particularly in the Indian setting [14,15].

Therefore, the present study was conducted to compare the functional outcomes of PHILOS plating and external fixation using K-wires with frames in patients with proximal humerus fractures. The primary objective of this study was to compare six-month Quick Disabilities of the Arm, Shoulder and Hand (QuickDASH) scores between PHILOS plating and external fixation using K-wires with frames [16]. Secondary objectives included comparison of fracture union, complications, shoulder range of motion, and perioperative outcomes. We hypothesized that PHILOS plating would be associated with better functional outcomes.

## Materials and methods

Study design and setting

This prospective comparative observational study was conducted at the Department of Orthopedics, Government Doon Medical College and associated Doon Hospital, Dehradun, Uttarakhand, over a period of 15 months (October 2024 to December 2025). The study aimed to compare the functional outcomes of PHILOS plating and external fixation using K-wires with frames in adult patients with proximal humerus fractures. The study was approved by the Institutional Ethics Committee of Government Doon Medical College, Dehradun (Ref No. GDMC/IEC/2024/25 dated 21/08/24). Written informed consent was obtained from all participants prior to enrolment.

Study population and sample size

A total of 60 patients with proximal humerus fractures were included in the study: 30 were treated with PHILOS plates (MEDPLUS, Ahmedabad, India) and 30 were treated with external fixation using K-wires and frames (MEDPLUS, Ahmedabad, India). The sample size was estimated using the formula n = 2(Zα + Zβ)²σ²/d², where n represents the required sample size per group, Zα was 1.96 for a two-sided 5% level of significance, Zβ was 0.84 for 80% power, σ was the assumed standard deviation, and d was the expected mean difference. An expected mean difference of 10 points in functional outcome scores was considered clinically meaningful based on the findings of Bamaniya and Panchnani, who reported an approximately 10-point difference in Constant-Murley scores between PHILOS plating and K-wire fixation groups [5]. The Constant-Murley score is a validated composite measure of shoulder pain, activities of daily living, range of motion, and strength [17]. Considering an assumed standard deviation of 14, the calculated sample size was approximately 30 patients per group after rounding to the nearest feasible whole number. Therefore, 60 patients, with 30 patients in each group, were included during the study period.

Eligibility criteria

Patients aged more than 18 years with closed proximal humerus fractures were eligible for inclusion. Patients with pathological fractures, open fractures, ipsilateral upper limb injuries or fractures, severe osteoporotic proximal humerus fractures, pregnancy, or comorbid conditions that rendered them unfit for surgery were excluded from the study.

Clinical and radiological evaluation

All patients underwent detailed clinical assessment, including history taking and physical examination. Radiological evaluation was performed using standard anteroposterior and scapular Y-view radiographs of the affected shoulder. Fractures were classified according to the Neer classification system, which divides the proximal humerus into four segments: the humeral head, greater tuberosity, lesser tuberosity, and humeral shaft [18]. A fracture fragment was considered displaced when angulation exceeded 45° or displacement was greater than 1 cm.

Treatment groups, surgical procedures, and follow-up

Patients were managed surgically using either PHILOS plating or external fixation with K-wires and frames according to the treating surgeon’s decision, fracture configuration, bone quality, and soft-tissue condition. Patients undergoing PHILOS plating constituted Group I, while those treated with external fixation constituted Group II. Treatment selection was based on the treating surgeon's clinical judgment after considering fracture configuration, bone quality, soft-tissue condition, and patient-related factors.

For PHILOS plating, surgery was performed under general anesthesia with the patient in a beach-chair position. A standard deltopectoral approach was used to expose the fracture site. Fracture fragments were reduced under direct visualization, with restoration of the humeral head-shaft alignment and tuberosity position. Temporary K-wires were used when required to maintain reduction. A PHILOS plate was positioned lateral to the bicipital groove and fixed to the humeral shaft using cortical screws and to the humeral head using multiple locking screws under fluoroscopic guidance. Screw position and fracture reduction were confirmed using anteroposterior and scapular Y-view fluoroscopy before wound closure.

For external fixation, surgery was performed under general anesthesia with the patient in a beach-chair position. Closed reduction was achieved under fluoroscopic guidance using traction and manipulation. Multiple percutaneous K-wires were inserted into the humeral head and proximal fracture fragments, avoiding penetration of the articular surface. Additional K-wires were inserted into the humeral shaft and connected externally using a frame construct to maintain reduction. Fluoroscopy was used to confirm fracture alignment, wire placement, and stability of the construct. Pin sites were dressed regularly, and patients were instructed regarding pin-site care.

Postoperatively, all patients received analgesics, wound or pin-site care, and a standardized rehabilitation protocol. The rehabilitation protocol was standardized for both groups and progressed according to pain tolerance and fixation stability. Pendulum and assisted shoulder exercises were initiated according to pain tolerance and fixation stability. Patients were followed up in the outpatient department at the second week, fourth week, third month, sixth month, and 12th month after surgery.

Outcome measures

Functional outcome was assessed using the QuickDASH questionnaire at the first, third, and sixth postoperative months [16]. The QuickDASH questionnaire consists of 11 items, each scored from 1 to 5. The total score was calculated using the formula: QuickDASH score = ((sum of responses/number of completed responses) - 1) × 25. Scores range from 0 to 100, where 0 represents no disability and 100 represents maximum upper limb disability. Lower QuickDASH scores therefore indicate better functional outcome. The primary outcome measure was the QuickDASH score at six months. Secondary outcome measures included radiological union, time to union, shoulder range of motion, operative duration, intraoperative blood loss, fluoroscopy exposure, length of hospital stay, and postoperative complications. Final functional outcome was categorized using the six-month QuickDASH score as excellent (0-20), good (21-40), fair (41-60), or poor (>60). For analysis of factors associated with outcome, excellent and good outcomes were grouped together, while fair and poor outcomes were grouped together.

Statistical analysis

Data were analyzed using IBM SPSS Statistics for Windows, Version 27.0 (IBM Corp., Armonk, USA). Categorical variables were expressed as frequency and percentage, while continuous variables were presented as mean ± standard deviation. Normality of continuous variables was assessed using the Kolmogorov-Smirnov test. Independent-samples t-test was used to compare normally distributed continuous variables between the two groups. Chi-square test, Yates-corrected Chi-square test, or Fisher’s exact test was used for comparison of categorical variables, as appropriate. Repeated-measures analysis of variance was used to evaluate changes in QuickDASH scores over time and to assess the effects of time, treatment group, and time × treatment group interaction. Partial η² was reported as the measure of effect size for repeated-measures analysis. A two-tailed p-value of less than 0.05 was considered statistically significant.

## Results

The PHILOS plating and K-wires with frames groups were comparable at baseline. The mean age was 49.67 ± 13.58 years and 48.87 ± 14.20 years, respectively (p = 0.824), while the mean time from injury to surgery was 5.37 ± 2.38 days and 5.02 ± 2.47 days (p = 0.577). Male patients comprised 18 (60.0%) and 17 (56.7%) of the patients in the two groups, respectively (p = 0.793). Right-sided fractures were observed in 17 (56.7%) patients in the PHILOS group and 16 (53.3%) patients in the K-wires group (p = 0.795). Controlled comorbidities were present in 10 (33.3%) and 11 (36.7%) patients, respectively (p = 0.787). No significant differences were observed between the groups for any baseline characteristic (Table 1).

**Table 1:** Baseline demographic and clinical characteristics of the study population. Independent-samples t-test was used for continuous variables, while Chi-square test was used for categorical variables. PHILOS: proximal humerus internal locking system; K-wire: Kirschner wire

Variable	PHILOS plating (n=30)	K-wires with frames (n=30)	Statistic	p-value
Age (years), mean ± SD	49.67 ± 13.58	48.87 ± 14.20	t = 0.223	0.824
Time from injury to surgery (days), mean ± SD	5.37 ± 2.38	5.02 ± 2.47	t = 0.561	0.577
Male sex, n (%)	18 (60.0)	17 (56.7)	χ² = 0.069	0.793
Right-sided fracture, n (%)	17 (56.7)	16 (53.3)	χ² = 0.067	0.795
Any controlled comorbidity, n (%)	10 (33.3)	11 (36.7)	χ² = 0.073	0.787

Falls on level ground were the most common mechanism of injury in both groups, occurring in 12 patients (40.0%) in the PHILOS plating group and 13 patients (43.3%) in the K-wires with frames group, followed by road traffic accidents in 11 patients (36.7%) and 10 patients (33.3%), respectively. The distribution of injury mechanisms was comparable between the groups (χ² = 0.088, p = 0.993). Regarding fracture pattern, three-part fractures were the most common in the PHILOS group, affecting 14 patients (46.7%), whereas two-part fractures predominated in the K-wires group, occurring in 15 patients (50.0%). Four-part fractures were observed in three patients (10.0%) in each group. No significant difference was found in the distribution of Neer fracture types between the groups (χ² = 0.297, p = 0.862), indicating comparable fracture characteristics at baseline (Table 2).

**Table 2:** Fracture characteristics and mechanism of injury. Chi-square test was used for comparison between groups. PHILOS: proximal humerus internal locking system; K-wire: Kirschner wire

Variable	Domain	PHILOS plating (n=30)	K-wires with frames (n=30)	Statistic	p-value
Mode of injury, n (%)	Fall on level ground	12 (40.0)	13 (43.3)	χ² = 0.088	0.993
	Road traffic accident	11 (36.7)	10 (33.3)		
	Fall from height	5 (16.7)	5 (16.7)		
	Work-related trauma	2 (6.7)	2 (6.7)		
Neer fracture type, n (%)	Two-part fracture	13 (43.3)	15 (50.0)	χ² = 0.297	0.862
	Three-part fracture	14 (46.7)	12 (40.0)		
	Four-part fracture	3 (10.0)	3 (10.0)		

The mean operative duration was significantly longer in the PHILOS plating group (82.58 ± 14.75 minutes) than in the K-wires with frames group (54.32 ± 11.65 minutes) (t = 8.234, p <0.001). Intraoperative blood loss was also significantly higher with PHILOS plating (176.50 ± 42.29 mL vs. 61.80 ± 21.40 mL; t = 13.255, p <0.001). Conversely, fluoroscopy exposure was significantly greater in the K-wires with frames group (27.90 ± 6.41 shots vs. 18.62 ± 5.17 shots; t = -6.169, p <0.001). Hospital stay was significantly longer among patients treated with PHILOS plating (5.71 ± 1.57 days vs. 4.42 ± 1.18 days; t = 3.611, p = 0.001). Patients in the PHILOS group achieved painless assisted shoulder movement earlier (4.80 ± 1.30 weeks vs. 5.63 ± 1.46 weeks; t = -2.342, p = 0.023). However, the time to active-assisted abduction >90° (8.30 ± 2.21 weeks vs. 9.40 ± 2.50 weeks; p = 0.074) and radiological union (13.09 ± 2.88 weeks vs. 13.82 ± 3.43 weeks; p = 0.375) was comparable between the groups (Table 3).

**Table 3:** Operative and recovery parameters. Independent-samples t-test was used for comparison between groups. PHILOS: proximal humerus internal locking system; K-wire: Kirschner wire

Variable	PHILOS plating (n=30)	K-wires with frames (n=30)	t-value	p-value
Operative duration (min), mean ± SD	82.58 ± 14.75	54.32 ± 11.65	8.234	<0.001
Intraoperative blood loss (mL), mean ± SD	176.50 ± 42.29	61.80 ± 21.40	13.255	<0.001
Fluoroscopy exposure (shots), mean ± SD	18.62 ± 5.17	27.90 ± 6.41	-6.169	<0.001
Hospital stay (days), mean ± SD	5.71 ± 1.57	4.42 ± 1.18	3.611	0.001
Time to painless assisted movement (weeks), mean ± SD	4.80 ± 1.30	5.63 ± 1.46	-2.342	0.023
Time to active-assisted abduction >90° (weeks), mean ± SD	8.30 ± 2.21	9.40 ± 2.50	-1.816	0.074
Time to radiological union (weeks), mean ± SD	13.09 ± 2.88	13.82 ± 3.43	-0.894	0.375

Radiological union rates were comparable between the two treatment groups throughout follow-up. Union by 12 weeks was achieved in 21 patients (70.0%) in the PHILOS plating group and 18 patients (60.0%) in the K-wires with frames group (χ² = 0.659, p = 0.417). By 16 weeks, union was observed in 27 patients (90.0%) and 25 patients (83.3%), respectively (χ² = 0.577, p = 0.448). At 20 weeks, union had occurred in 29 patients (96.7%) treated with PHILOS plating and 28 patients (93.3%) treated with K-wires with frames (χ² = 0.351, p = 0.554). Delayed union was noted in one patient (3.3%) in the PHILOS group and two patients (6.7%) in the K-wires group, while no cases of non-union were observed in either group. None of the differences in radiological union outcomes reached statistical significance (Table 4).

**Table 4:** Radiological union outcomes. Chi-square test was used for comparison between groups, except for non-union because no events occurred in either group. PHILOS: proximal humerus internal locking system; K-wire: Kirschner wire

Variable	PHILOS plating (n=30)	K-wires with frames (n=30)	χ²	p-value
Union by 12 weeks, n (%)	21 (70.0)	18 (60.0)	0.659	0.417
Union by 16 weeks, n (%)	27 (90.0)	25 (83.3)	0.577	0.448
Union by 20 weeks, n (%)	29 (96.7)	28 (93.3)	0.351	0.554
Delayed union, n (%)	1 (3.3)	2 (6.7)	0.351	0.554
Non-union	0	0	-	-

Functional outcomes improved progressively in both groups during follow-up. At one month, the mean QuickDASH score was lower in the PHILOS plating group (58.70 ± 11.53) compared with the K-wires with frames group (63.90 ± 9.82), although the difference was not statistically significant (t = -1.883, p = 0.065). At three months and six months, the PHILOS group demonstrated significantly better functional outcomes, with lower QuickDASH scores of 31.80 ± 9.88 and 16.89 ± 8.48, respectively, compared with 37.60 ± 8.11 and 22.59 ± 8.54 in the K-wires group (t = -2.485, p = 0.016 and t = -2.595, p = 0.012, respectively). At six months, shoulder range of motion was significantly better in the PHILOS plating group, with greater forward flexion (142.60 ± 18.40° vs. 131.76 ± 21.65°; t = 2.090, p = 0.041) and abduction (136.87 ± 20.15° vs. 124.70 ± 22.51°; t = 2.206, p = 0.031). Although external rotation (48.21 ± 10.60° vs. 43.12 ± 11.76°; p = 0.084) and internal rotation scores (7.79 ± 1.39 vs. 7.07 ± 1.55; p = 0.064) were higher in the PHILOS group, the differences were not statistically significant (Table 5).

**Table 5:** Functional outcomes at follow-up. Independent-samples t-test was used for comparison between groups. QuickDASH: Quick Disabilities of the Arm, Shoulder and Hand; PHILOS: proximal humerus internal locking system; K-wire: Kirschner wire

Variable	PHILOS plating (n=30)	K-wires with frames (n=30)	t-value	p-value
QuickDASH at one month, mean ± SD	58.70 ± 11.53	63.90 ± 9.82	-1.883	0.065
QuickDASH at three months, mean ± SD	31.80 ± 9.88	37.60 ± 8.11	-2.485	0.016
QuickDASH at six months, mean ± SD	16.89 ± 8.48	22.59 ± 8.54	-2.595	0.012
Forward flexion (°), mean ± SD	142.60 ± 18.40	131.76 ± 21.65	2.090	0.041
Abduction (°), mean ± SD	136.87 ± 20.15	124.70 ± 22.51	2.206	0.031
External rotation (°), mean ± SD	48.21 ± 10.60	43.12 ± 11.76	1.759	0.084
Internal rotation score, mean ± SD	7.79 ± 1.39	7.07 ± 1.55	1.886	0.064

QuickDASH scores improved significantly over time in the overall study population, with a large time effect, F(1.565, 90.770) = 1958.187, p <0.001, partial η² = 0.971. The overall mean QuickDASH score across follow-up visits was significantly lower in the PHILOS plating group than in the K-wires with frames group, F(1, 58) = 5.774, p = 0.019, partial η² = 0.091. However, the time × treatment group interaction was not significant, F(1.565, 90.770) = 0.113, p = 0.845, partial η² = 0.002, indicating that the pattern of improvement over time was similar in both groups (Table 6).

**Table 6:** Repeated-measures analysis of QuickDASH scores over time in the overall study population (N=60). ^†^Greenhouse-Geisser correction was applied because Mauchly’s test indicated a violation of the sphericity assumption. Repeated-measures analysis of variance was used to assess the effects of time, treatment group, and the time × treatment group interaction. QuickDASH: Quick Disabilities of the Arm, Shoulder and Hand; df: degrees of freedom

Effect	F statistic, df	p-value	Partial η²
Time^†^	F(1.565, 90.770) = 1958.187	<0.001	0.971
Treatment group	F(1, 58) = 5.774	0.019	0.091
Time × treatment group interaction^†^	F(1.565, 90.770) = 0.113	0.845	0.002

Overall complication rates were identical in both groups, with eight patients (26.7%) in each group experiencing at least one complication (χ² = 0.000, p = 1.000). Excellent or good functional outcomes were achieved in 24 patients (80.0%) in the PHILOS plating group and 20 patients (66.7%) in the K-wires with frames group, whereas fair or poor outcomes were observed in six patients (20.0%) and 10 patients (33.3%), respectively. However, the overall distribution of final functional outcomes did not differ significantly between the groups (χ² = 2.054, p = 0.561) (Table 7).

**Table 7:** Complications and final functional outcome. Chi-square test was used for comparison between groups. PHILOS: proximal humerus internal locking system; K-wire: Kirschner wire

Variable	PHILOS plating (n=30)	K-wires with frames (n=30)	χ²	p-value
At least one complication, n (%)	8 (26.7)	8 (26.7)	0.000	1.000
Excellent outcome, n (%)	6 (20.0)	3 (10.0)	2.054	0.561
Good outcome, n (%)	18 (60.0)	17 (56.7)		
Fair outcome, n (%)	5 (16.7)	8 (26.7)		
Poor outcome, n (%)	1 (3.3)	2 (6.7)		

Fracture severity was significantly associated with final functional outcome (χ² = 9.393, p = 0.009). Excellent or good outcomes were achieved in 25 patients (89.3%) with two-part fractures, 17 patients (65.4%) with three-part fractures, and two patients (33.3%) with four-part fractures, indicating poorer outcomes with increasing fracture complexity. The presence of controlled comorbidities was associated with less favorable outcomes (χ² = 4.331, p = 0.037). Excellent or good outcomes were observed in 32 patients (82.1%) without comorbidities compared with 12 patients (57.1%) with comorbidities. Complications were also significantly associated with poorer functional outcomes (χ² = 9.764, p = 0.002). Excellent or good outcomes occurred in 37 patients (84.1%) without complications versus seven patients (43.8%) among those who developed complications (Table 8).

**Table 8:** Factors associated with poor functional outcome. Chi-square test was used for analysis.

Variable	Domain	Excellent/Good, n (%)	Fair/Poor, n (%)	χ²	p-value
Neer fracture type	Two-part fracture	25 (89.3)	3 (10.7)	9.393	0.009
	Three-part fracture	17 (65.4)	9 (34.6)		
	Four-part fracture	2 (33.3)	4 (66.7)		
Any controlled comorbidity	Absent	32 (82.1)	7 (17.9)	4.331	0.037
	Present	12 (57.1)	9 (42.9)		
Any complication	No	37 (84.1)	7 (15.9)	9.764	0.002
	Yes	7 (43.8)	9 (56.3)		

## Discussion

The present study compared functional and radiological outcomes of PHILOS plating and external fixation using K-wires with frames in patients with proximal humerus fractures. Baseline characteristics were comparable between the two groups, with no significant differences in age, sex distribution, fracture side, time from injury to surgery, or comorbidity status. This baseline comparability reduces potential confounding and strengthens the validity of the comparison between the two treatment groups. This comparability minimizes selection bias and allows a more reliable assessment of treatment-related outcomes. Similar baseline distributions have been reported in previous comparative studies evaluating surgical options for proximal humerus fractures [19-21].

The pattern of injury and fracture morphology were also similar between groups. Falls on level ground represented the most common mechanism of injury, followed by road traffic accidents. Two-part and three-part fractures accounted for the majority of cases, whereas four-part fractures were relatively uncommon. These findings are consistent with previous studies reporting that displaced two-part and three-part fractures constitute the largest proportion of surgically treated proximal humerus fractures [14,22-24]. The comparable fracture distribution between groups ensured that differences in outcomes were not attributable to variations in fracture severity.

A major finding of the present study was the difference in operative characteristics between the two treatment modalities. PHILOS plating required significantly longer operative time, greater intraoperative blood loss, and longer hospitalization. These observations are expected because PHILOS plating involves open reduction and internal fixation with greater soft tissue exposure. Similar findings have been reported by Kothari et al., Wang et al., and De Joseph et al., who demonstrated increased operative complexity with locking plate fixation [19-21]. Conversely, fluoroscopy exposure was significantly higher in the external fixation group, reflecting the dependence on image-guided closed reduction and percutaneous wire placement, a finding also reported in minimally invasive fixation studies [11,21].

Despite increased operative morbidity, PHILOS plating facilitated earlier painless shoulder mobilization. Patients treated with PHILOS plating achieved painless assisted movement significantly earlier than those managed with external fixation. Earlier mobilization may be attributed to the greater mechanical stability provided by locking plate constructs, allowing more confident rehabilitation protocols. Previous studies have similarly emphasized the advantages of stable fixation in promoting early functional recovery [19,24,25].

Radiological outcomes were excellent in both groups. Union rates progressively increased during follow-up and exceeded 90% by 16 weeks in both treatment arms. Delayed union was uncommon, and no patient developed non-union. Furthermore, the mean time to radiological union was comparable between groups. These findings suggest that both PHILOS plating and external fixation can provide adequate fracture stability for successful healing when applied appropriately. Comparable union rates have been reported in studies evaluating locking plates and external fixation techniques [10,19,21,24].

Functional outcomes favored PHILOS plating. Although the difference in QuickDASH scores at one month did not reach statistical significance, patients treated with PHILOS plating demonstrated significantly lower QuickDASH scores at both three and six months. In addition, forward flexion and abduction at six months were significantly better in the PHILOS group. These findings indicate superior medium-term functional recovery with PHILOS plating and are consistent with previous reports demonstrating improved shoulder function following locking plate fixation [19,21,22,25,26]. Although statistically significant improvements in QuickDASH scores were observed with PHILOS plating, the clinical relevance of these differences should be interpreted alongside individual patient characteristics and treatment goals.

Repeated-measures analysis showed significant improvement in QuickDASH scores over time in the overall study population, confirming that both surgical techniques were effective in restoring shoulder function. However, the absence of a significant time × treatment interaction suggests that the pattern of recovery was similar in both groups, despite the PHILOS group achieving better absolute functional scores.

Complication rates were identical in both groups, with approximately one-quarter of patients experiencing at least one complication. Importantly, the overall distribution of final functional outcomes did not differ significantly between treatment modalities. Although a higher proportion of patients in the PHILOS group achieved excellent outcomes, both techniques produced predominantly good or excellent results. Similar observations have been reported by Kothari et al. and other comparative studies, suggesting that both methods are viable treatment options when appropriately selected [21,24,26].

An important observation was the influence of fracture complexity, comorbidity status, and complications on final outcome. Patients with two-part fractures achieved the best results, whereas outcomes progressively worsened with increasing fracture complexity. Likewise, patients with comorbidities and those who developed complications were significantly more likely to have fair or poor outcomes. These findings agree with previous literature identifying fracture severity, systemic health status, and postoperative complications as important determinants of functional recovery [27-29].

Overall, the present study demonstrates that both PHILOS plating and external fixation using K-wires with frames achieve satisfactory fracture union and acceptable functional recovery. However, PHILOS plating provides superior shoulder function, earlier painless mobilization, and better medium-term QuickDASH outcomes, albeit at the cost of longer surgery, increased blood loss, and prolonged hospitalization. These findings support PHILOS plating as the preferred option for suitable patients, while external fixation remains a valuable minimally invasive alternative in selected cases.

This study has several limitations. First, it was a single-center prospective comparative observational study with a relatively small sample size, which may limit its generalizability. Second, treatment allocation was based on surgeon decision and fracture characteristics rather than randomization; therefore, residual selection bias cannot be excluded despite comparable baseline characteristics. Third, follow-up functional assessment was primarily limited to six months, and longer-term outcomes such as late implant-related complications, avascular necrosis, and persistent stiffness may not have been fully captured. In addition, no multivariable adjustment was performed for potential confounding factors, and multiple statistical comparisons may have increased the risk of type I error. Finally, the study did not include blinding of outcome assessment, which may have introduced observer-related bias. Future multicenter randomized controlled trials with larger sample sizes and longer follow-up are needed to confirm these findings and evaluate long-term outcomes, including avascular necrosis, implant failure, and sustained shoulder function.

## Conclusions

Both PHILOS plating and external fixation using K-wires with frames provided satisfactory radiological union and functional recovery in proximal humerus fractures. PHILOS plating resulted in significantly better functional outcomes, including lower QuickDASH scores, earlier painless shoulder mobilization, and greater shoulder range of motion at follow-up. However, it was associated with longer operative duration, greater blood loss, and longer hospital stay. Union rates and complication rates were comparable between the groups. Fracture complexity, comorbidities, and postoperative complications were significant predictors of poorer outcomes. Patients treated with PHILOS plating, thus, demonstrated better functional outcomes. However, treatment selection should be individualized according to fracture complexity, bone quality, and patient factors.

## References

[1] Rudran B, Little C, Duff A, Poon H, Tang Q (2022). Proximal humerus fractures: anatomy, diagnosis and management. Br J Hosp Med (Lond).

[2] Iglesias-Rodríguez S, Domínguez-Prado DM, García-Reza A, Fernández-Fernández D, Pérez-Alfonso E, García-Piñeiro J, Castro-Menéndez M (2021). Epidemiology of proximal humerus fractures. J Orthop Surg Res.

[3] Patel AH, Wilder JH, Ofa SA, Lee OC, Savoie FH 3rd, O'Brien MJ, Sherman WF (2022). Trending a decade of proximal humerus fracture management in older adults. JSES Int.

[4] Chelli M, Gasbarro G, Lavoué V, Gauci MO, Raynier JL, Trojani C, Boileau P (2022). The reliability of the Neer classification for proximal humerus fractures: a survey of orthopedic shoulder surgeons. JSES Int.

[5] Bamaniya D, Panchnani S (2019). Comparative evaluation of functional outcomes of proximal humerus fractures treated with PHILOS plate and K-wire fixation. Int J Orthop Sci.

[6] Launonen AP, Sumrein BO, Reito A (2019). Operative versus non-operative treatment for 2-part proximal humerus fracture: a multicenter randomized controlled trial. PLoS Med.

[7] Large TM, Adams MR, Loeffler BJ, Gardner MJ (2019). Posttraumatic avascular necrosis after proximal femur, proximal humerus, talar neck, and scaphoid fractures. J Am Acad Orthop Surg.

[8] Doshi C, Sharma GM, Naik LG, Badgire KS, Qureshi F (2017). Treatment of proximal humerus fractures using PHILOS plate. J Clin Diagn Res.

[9] Foruria AM (2023). Plate fixation of proximal humerus fractures: how to get it right and future directions for improvement. Curr Rev Musculoskelet Med.

[10] Blonna D, Assom M, Bellato E, Pisanu G, Greco V, Marmotti A, Rossi R (2019). Outcomes of 188 proximal humeral fractures treated with a dedicated external fixator with follow-up ranging from 2 to 12 years. J Bone Joint Surg Am.

[11] Fink Barnes L, Parsons BO, Flatow EL (2015). Percutaneous fixation of proximal humeral fractures. JBJS Essent Surg Tech.

[12] Boadi PJ, Da Silva A, Mizels J, Joyce CD, Anakwenze OA, Klifto CS, Chalmers PN (2024). Intramedullary versus locking plate fixation for proximal humerus fractures: indications and technical considerations. JSES Rev Rep Tech.

[13] Nowak LL, Davis AM, Mamdani M, Beaton D, Kennedy C, Schemitsch EH (2019). A systematic review and standardized comparison of available evidence for outcome measures used to evaluate proximal humerus fracture patients. J Orthop Trauma.

[14] Pandey R, Raval P, Manibanakar N, Nanjayan S, McDonald C, Singh H (2023). Proximal humerus fracture(s): a review of current practice. J Clin Orthop Trauma.

[15] Slobogean GP, Johal H, Lefaivre KA (2015). A scoping review of the proximal humerus fracture literature. BMC Musculoskelet Disord.

[16] Beaton DE, Wright JG, Katz JN (2005). Development of the QuickDASH: comparison of three item-reduction approaches. J Bone Joint Surg Am.

[17] Constant CR, Murley AH (1987). A clinical method of functional assessment of the shoulder. Clin Orthop Relat Res.

[18] Neer CS 2nd (1970). Displaced proximal humeral fractures. I. Classification and evaluation. J Bone Joint Surg Am.

[19] Wang M, Wang X, Cai P, Guo S, Fu B (2023). Locking plate fixation versus intramedullary nail fixation for the treatment of multifragmentary proximal humerus fractures (OTA/AO type 11C): a preliminary comparison of clinical efficacy. BMC Musculoskelet Disord.

[20] De Joseph J, Gandhi P A, Padhi BK (2024). PHILOS (proximal humerus internal locking system) plating vs MultiLoc nailing in proximal humerus fracture: systematic review and meta-analysis of radiological and functional outcomes. Cureus.

[21] Kothari C, Pundkare G, Desai A (2025). Comparative analysis of proximal humerus internal locking system plating and external fixation in proximal humerus fracture management: a prospective study on functional outcomes. Int J Res Orthop.

[22] Jain A, Bhalla N, Gangwar D (2025). Functional outcome of proximal humerus internal locking system (PHILOS) plating in proximal humerus fractures. Cureus.

[23] Sathavu PP, Kothimbakkam PK, Murugan PB, Mohideen S, Murugesan V, Ranganathan T (2025). Functional and radiological outcome of proximal humerus fractures treated with proximal humerus internal locking osteosynthesis system plating - a prospective study. J Orthop Case Rep.

[24] Thakur P, Mahmood ST, Shakya M, Chatterji G, Bindal A, Kathal A, Agarwal V (2024). Functional outcome of proximal humeral fractures treated with PHILOS plate in adults. Int J Orthop Traumatol.

[25] Kale A, Sharma P, Kuity K, Muneer MT, Verma A, Kaneria S (2024). A study of functional outcome and assessment of role of proximal humerus internal locking system (PHILOS) plating in elderly population with proximal humerus fracture: a case series. J Orthop Case Rep.

[26] Saraf A, Bishnoi S, Parakh NK, Kumar SK, Chaudhary A, Reddy KD (2025). Clinical and functional outcomes of proximal humerus internal locking system plate fixation in proximal humerus fractures: a short-term follow-up. J Orthop Case Rep.

[27] Reito A, Launonen A, Tootsi K (2025). Prediction of patient-reported outcomes after proximal humerus fractures in elderly patients does not appear to be a credible option: a secondary analysis of two randomized controlled trials. Bone Joint Res.

[28] Hohmann E, Keough N, Glatt V, Tetsworth K (2023). Surgical treatment of proximal humerus fractures: a systematic review and meta-analysis. Eur J Orthop Surg Traumatol.

[29] Bergdahl C, Wennergren D, Ekelund J, Möller M (2020). Mortality after a proximal humeral fracture. Bone Joint J.

